# Data on samara morphology and wind dispersal in the invasive tree *Ailanthus altissima*

**DOI:** 10.1016/j.dib.2019.104521

**Published:** 2019-09-18

**Authors:** Greg Planchuelo, Pablo Catalán, Juan A. Delgado, Antonio Murciano

**Affiliations:** a Department of Ecology, Technische Universität Berlin, Germany; b Biosciences, College of Life and Environmental Sciences, University of Exeter, UK; c Department of Biodiversity, Ecology and Evolution, Universidad Complutense, Spain

**Keywords:** *Ailanthus altissima*, Diaspore morphology, Samara, Three-dimensional structure, Wind dispersal potential

## Abstract

The data presented in this paper is supporting the research article “Estimating wind dispersal potential in *Ailanthus altissima*: The need to consider the three-dimensional structure of samaras” [1]. We analyzed the estimation of samara's wind dispersal potential through a group of morphological variables that succeed in describing the three-dimensional nature of samaras. We present here a dataset containing 8 morphological variables of 200 samaras belonging to 5 different individuals of the invasive tree *Ailanthus altissima* (Mill.) Swingle. Additionally, we present the average descent velocity of each of the samaras, which was recorded by releasing 5 times each samara under controlled and reproducible conditions. The data set is structured in a single spreadsheet where we also included the samara and the individual identity code of the tree.

**Table:** Specification table

Subject area	Biology
More specific subject area	Dispersal ecology
Type of data	Figure and table
How data was acquired	Seed dispersal experiment
Data format	Raw
Experimental factors	Damaged samaras were discarded from the experiment. Undamaged samaras were used for this experiment and were not modified in any way. Samaras were dropped from 2 m high in still air to obtain descent velocity measurements.
Experimental features	Average descent velocity of each samara (5 replicates), samara mass and morphometric variables (from a frontal view: frontal area, frontal perimeter, width, length; and from a side view: side perimeter and side height, Fig. 1). A total of 200 samaras belonging to five different individuals of *Ailanthus altissima*.
Data source location	Madrid, Spain.
Data accessibility	Open access in the Mendeley online repository: G. Planchuelo, P. Catalán, J.A. Delgado, A. Murciano, Data on samara morphology and wind dispersal in the invasive tree *Ailanthus altissima*, Mendeley Data (2019), https://doi.org/10.17632/z43kbprcg9.1
Related research article	G. Planchuelo, P. Catalán, J.A. Delgado, A. Murciano, Estimating wind dispersal potential in *Ailanthus altissima*: The need to consider the three-dimensional structure of samaras, Plant Biosyst. 151 (2017) 316–322, https://doi.org/10.1080/11263504.2016.1174170.

**Table:** 

**Value of the data** • Average descent velocity (m s^−1^) is a measure of wind dispersal potential. The assumption is that slower fall velocities are related to higher dispersal potential of each individual seed. • The inclusion of morphological variables related to the three-dimensional structure of the samara enables a better understanding of the relationship between samara morphology and dispersal. • Considering *Ailanthus altissima* is an invasive species, recorded dispersal potential could be compared with the records from other areas, including its native range, aiming to understand its relation to invasion potential. • The inclusion of morphological traits from samaras from different parent trees shows intraindividual variation in samara morphology and its consequences in terms of dispersal ability.

## 1 Data

**Fig. 1:**
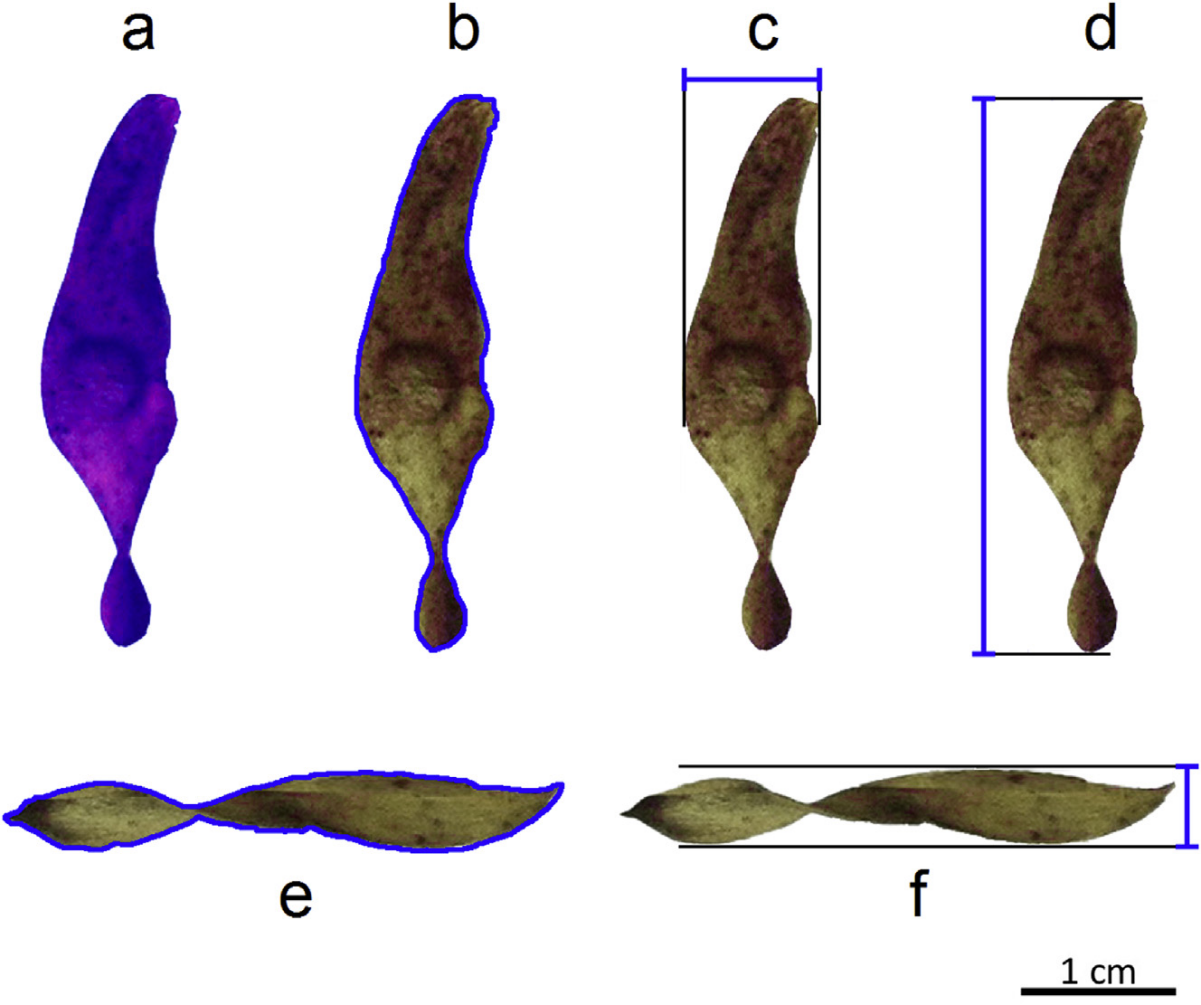
Some of the morphological measurements taken (in blue) for each samara: (a) frontal area; (b) frontal perimeter; (c) width; (d) length; (e) side perimeter; (f) side height.

The dataset we present contains information of the average descent velocity and the morphology of samaras of *Ailanthus altissima* trees growing spontaneously in an urban area out of its native range. The dataset contains measurements performed on 200 samaras belonging to 5 different trees (40 samaras per tree). Samaras are classified according to the tree they belong to. The average descent velocity along with the mass and 7 morphometric traits have been measured for each samara. Average descent velocity was calculated averaging 5 independent measurements for each samara. Morphometric traits are: frontal area, frontal perimeter, width, length, side area, side perimeter and side height (Fig. 1). Frontal and side areas refer to the surface projected by the samara when observed from a frontal and side view, respectively. The frontal and side perimeters are the total length measured around the outline of the samara when observed from a frontal and side view, respectively. The length expresses the distance from end to end along the longitudinal axis of the samara. The width and the side height are the longest distance running perpendicular to the samaras' longitudinal axis while observing the samara from a frontal view and a side view, respectively. Morphometric traits were measured with Adobe Photoshop CS6 and ImageJ v1.47 by processing photographs of the samaras taken from a side and a frontal view along a ruler for calibration.

The data presented in this paper was used for analyses in Planchuelo et al. [1].

## 2 Experimental design, materials and methods

Fieldwork was carried out on UCM Campus in the urban area of Madrid, Spain. We randomly selected five spontaneous female *A. altissima* trees. We presented data on 8 morphological variables of 40 ripened samaras from each of them. We recorded the mass of the samara and morphometric traits measured on a frontal view of the samara (frontal area, frontal perimeter, width and length), but we also measured variables on a side view of the samara (side area, side perimeter, side height) to obtain information of the three-dimensional structure of the samara (Fig. 1). These variables have been shown to relate to the wind dispersal capabilities of the samaras: Frontal area, frontal perimeter and side area are related to the surface area of the samara, which has a direct impact on the flying capabilities of the samaras [2], [3]. The width, length and side perimeter can also have an effect on wind dispersal, as they are related to the autorotation capacity of the samara [4]. Side perimeter and side height are related respectively to the magnitude of a samara's spiral twist and its deviation from the samara's axis, which have been shown to affect autorotation capacity and hence be related to the flying potential of the samaras [4], [5].

Additionally, the descent velocity (m s^−1^) of each samara was calculated by measuring the time it took to fall through a distance of 2 m in an airtight and sealed chamber [6]. Measurements were repeated five times and an average was calculated after verifying the consistency and repeatability of the data by means of an intraclass correlation coefficient (ICC). Throughout the course of this study, samaras were not painted, colored, written on, modified or altered in any way.

## Conflict of Interest

The authors declare that they have no known competing financial interests or personal relationships that could have appeared to influence the work reported in this paper.
